# The role of skin self-examination at the Swiss skin cancer day

**DOI:** 10.1186/s12913-014-0581-6

**Published:** 2014-11-19

**Authors:** Nina Badertscher, Muriel Meier, Thomas Rosemann, Ralph Braun, Antonio Cozzio, Brigitte Tag, Michel Wensing, Ryan Tandjung

**Affiliations:** Institute for Primary Care, University of Zurich, University Hospital of Zurich, Pestalozzistrasse 24, 8091 Zürich, Switzerland; Department of Dermatology, University Hospital of Zurich, Rämistrasse 100, 8091 Zürich, Switzerland; Institute of Law, University of Zurich, Freiestrasse 15, 8032 Zürich, Switzerland; Radboud University Nijmegen Medical Centre, Post 114, Postbus 9101, 6500 HB, Nijmegen, The Netherlands

**Keywords:** Skin cancer, Prevention, Skin self-examination, Swiss skin cancer day

## Abstract

**Background:**

The rising incidence of melanoma – Switzerland has the highest incidence in Europe - is a major public health challenge. Swiss dermatologist introduced the “Swiss Skin Cancer Day” (SSCD) in 2006, which provides skin cancer screening at no costs. The aim of the study was to describe the participating subjects and their motivation and investigate factors influencing the probability of a clinical diagnosis of skin malignancy.

**Methods:**

150 dermatologists were involved in the SSCD in May 2012. Dermatologists were not remunerated. Participants had the opportunity to show a single skin lesion to a dermatologist at no cost. A questionnaire for each participating subject collected data about subjects’ age, sex, risk factors and reason for encounter; furthermore the dermatologist noted down clinical diagnosis and further management. We used descriptive statistics to report characteristics of participants and skin lesions. We built two multiple logistic regression models, one regarding the clinical diagnosis of skin malignancy and one regarding the further management.

**Results:**

5266 subjects (55.6% female) were assessed; in 308 (5.8%) participants a clinical diagnosis of skin malignancy was found. In 1732 participants (32.9%) a clinical follow up or an excision was recommended. In the multiple logistic regression model age, sex, skin phototype and the reason for participation at the SSCD were found as significant risk factors regarding the clinical diagnosis of skin malignancy. Participants with skin cancer risk factors were more likely to get a clinical follow up recommended even if the clinical diagnosis was benign.

**Conclusion:**

A self-perceived suspicious lesion was the strongest predictor for a clinical diagnosis of skin malignancy at the SSCD. This suggests that skin self-examination might also work in general population. Future research should focus on better access to a specialist in case a suspicious skin lesion was discovered. Safety and quality of the SSCD should be further investigated, especially concerning the discrepancy between the low number of malignant lesions and the high quantity of participants where further clinical examinations or interventions were recommended.

**Electronic supplementary material:**

The online version of this article (doi:10.1186/s12913-014-0581-6) contains supplementary material, which is available to authorized users.

## Background

The rising incidence of melanoma over the past decades is a major public health challenge [1,2]. Switzerland (26.8 cases per 100’000 in male and 25.4 in female population; European age-standardised rates) is among the countries with the highest incidence [3]. Clinical examination of skin does not require expensive technical equipment and early detection of melanoma has a huge impact on prognosis [4], thus cutaneous melanoma seems to be prone to preventive services. While screening in high-risk patients was shown to reduce mortality and to be cost-effective [5,6], the evidence on effectiveness of screening programmes in general population remains inconclusive [7-12]. German studies on a pilot in Schleswig-Holstein and a consecutively introduced nationwide screening programme produced promising results [10,13,14]. Compared to the pre-screening era the mortality of melanoma was reduced (from 1.9/100’000 to 1.0 in men and from 1.4 to 0.7 in women), while the mortality was stable in four adjacent regions. Studies about the role of skin self-examination (SSE) produced promising results: some studies showed that SSE reduces tumor thickness and melanoma incidence in high-risk patients [15-18].

The Swiss healthcare system has no mandatory gate keeping system. Dermatologists are working in outpatient clinics as well as in private practices. Even though many patients choose an insurance model without gatekeeping, a direct consultation with a dermatologist is hardly possible and a referral from primary care physicians is usually mandatory. Therefore a suspicious lesion in SSE might have unnecessary barriers with a contact to a primary care physician and a possible secondary referral to a dermatologist, since many primary care physicians also feel rather insecure concerning the diagnosis of melanoma [19].

Switzerland lacks a nationally coordinated cancer screening strategy, some prevention investigation such as a colonoscopy screening after the age of 50 have recently been taken in the remuneration scheme of insurance companies. A general skin cancer screening has not been implemented so far, but local campaigns started in the late 80ies. The evaluation of a skin cancer campaign in the year 2000 showed an important role of such campaigns in the early detection of melanoma [20]. In 2006 a national and annual prevention programme was introduced, when Switzerland joined the European prevention campaign “Euromelanoma Day”, where nowadays dermatologists of over 20 European countries participate. The prevention campaign contains both: a public health information campaign through mass media about sun protection and the importance of an early detection of melanoma and a special event, where anyone can participate and will be examined by participating dermatologists for free [21-23]. In Switzerland the prevention campaign is called “Swiss Skin Cancer Day” (SSCD). Nowadays, the SSCD is a multifaceted campaign with different activities, such as public lectures about skin cancer, risk factors for melanoma and the importance of an early detection of melanoma; a media campaign concerning the importance of sun protection and several other public events. The main focus is on primary prevention, but among these activities an opportunity of a free single skin lesion check is offered to the public, which has to effects: 1) raising awareness for the importance of primary prevention concerning skin cancer and 2) a possible early detection of skin cancer (secondary prevention). In times of scarce health professionals and rising health care costs an implementation of a general screening program might not be feasible. Therefore an event such as the “Swiss Skin Cancer Day”, “Euromelanoma Day” or “Melanoma Monday” in the US might also be effective [21,24-27].

However two facts might limit the effect of such an event: During the SSCD resources do not allow a full body exam or the standardized use of dermoscopy, but only an investigation of a single lesion. And second, prevention campaigns are likely to reach a younger and more health-conscious population and therefore might not aim at the population most likely to profit [28,29]. Former SSCD participants stated different reasons for their participation: while some subjects had a specific lesion they wanted to show to a dermatologist, others just wanted to use the opportunity of a free skin check-up. The aim of the study was to describe the participating subjects and their motivation and investigate factors influencing the probability of a suspicion of malignancy.

## Methods

### Swiss skin cancer day

Among the activities of the SSCD, on special scheduled slots dermatologists in private practices as well as outpatient clinics offer the possibility to evaluate skin lesions of participants. Dermatologists in private practices and Clinics of Dermatology had the freedom to participate or not; participation was not remunerated. On three afternoons in May 2012 (7^th^, 9^th^ and 12^th^) over 150 dermatologists in the French and German speaking parts of Switzerland offered such opportunities, all ten dermatology clinics and 63 (out of 384) dermatologists in private practice contributed to the SSCD 2012. For the patients participation was free of charge and no special appointment was necessary. Participants had the possibility to show a single skin lesion only; the logistic challenges – mainly time constraints – did not allow a full body scan. A dermoscopic investigation was allowed, although not standardized and due to time constraints not routinely offered and not documented.

### Assessment instruments

We created a paper-based questionnaire collecting anonymous data about patient characteristics (sex, age, nationality), main reason for participation on the SSCD (specific lesion vs. general check-up vs. skin cancer of friends), risk factors (Fitzpatrick phototype, over 100 nevi, skin cancer in family), and clinical diagnosis based on the examination and recommendation for further management. The further management was categorised into three domains: 1) no further step necessary 2) clinical observation and 3) excision or biopsy recommended. The questionnaire was completely filled by the participating dermatologists. Data management was centrally organized at the Institute for Primary Care of the University of Zurich. A questionnaire is provided in Additional file 1.

We considered lentigo maligna, melanoma, basal cell carcinoma, squamous cell carcinoma and “other malignant diagnosis” as malignant.

### Statistics

We used descriptive statistics to report characteristics of participants and skin lesions. To compare male and female participants we used chi-square testing regarding the participants’ characteristics. We created a multiple logistic regression to assess participants’ characteristics (age, sex, reason for participation, more than 100 naevi, skin phototype and family skin cancer history) associated with a higher risk of a clinical diagnosis of skin malignancy. Since we were specifically interested whether self-screening was efficient, we created a dichotomous variable (1 = self-perceived lesion, 0 = all other). We performed analyses using Stata® Version 12.1 (Stata Corporation, College Station, TX 77845, USA, www.stata.com). We regarded a p < 0.01 as statistical significant.

We created a second multiple regression model concerning further management. We excluded the participants with clinical malignant diagnosis. We grouped the participants where a biopsy or excision and where a clinical observation was recommended (biopsy or clinical observation necessary = 1, no further investigation necessary = 0). Number of naevi, skin phototype, positive family history and age were included in the regression modell as influence factors.

### Ethics approval

Under current ethical guidelines of the Swiss Academy of Medical Sciences [30] this study collecting completely anonymous data did not need formal ethical approval. Data was treated confidentially. On a general information letter of the SSCD activities participants were informed that data collected would be analysed in anonymized form.

## Results

During the three afternoons in May 2012, a total of 5266 subjects were screened (44.4% male, 55.6% female), the average age was 51.2 years (SD 19.27) for male, and 48.1 years (SD 18.47) for female subjects (p = 0.224). The prevalence of skin types was: Type I 323 (6.1%), Type II 2354 (44.7%), Type III 1734 (33.0%), Type IV 257 (4.9%), Type V 45 (0.9%), Type VI 7 (0.1%), 546 entries were missing. In the estimation of naevi 4090 (77.7%) had less than 100 naevi, 613 (11.6%) more than 100; 563 data are missing. 379 (7.2%) have a family history of skin cancer. We asked subjects for their reason to participate. The majority of 3178 subjects (60.4%) declared that they wanted to use the possibility of a free check-up of the skin. 1530 (29.1%) were using the possibility to show a self-perceived suspicious skin lesion, 87 (1.7%) had skin cancer cases in their entourage, 276 (5.25%) were sent by their partners, 188 (3.6%) stated different or no reasons. In Table 1 the risk factors are listed in female and male participants.

**Table 1:** **Characteristics of female and male participants**

	**Female**	**Male**	**Significance level**
**Age in years** **(mean,** **SD)**	48.1 (18.47)	51.2 (19.27)	p = 0.224
**Skin phototype** **(%)**			P < 0.001
**I**	8.37	4.89	
**II**	51.22	48.18	
**III**	33.92	40.40	
**IV**	5.25	5.71	
**V**	1.06	0.77	
**VI**	0.19	0.05	
**More than 100 naevi** **(%)**	11.31	15.23	p < 0.001
**Family history of skin cancer** **(%)**	8.27	5.87	p = 0.001
**Reason for participation** **(%)**			p < 0.001
**Checkup**	63.15	57.03	
**Suspicious skin lesion**	29.47	28.62	
**Skin cancer of friends**	1.54	1.75	
**Sent by partner**	9.55	1.81	
**Other**	3.26	3.83	

Numbers indicated age in years and all other variable indicate frequencies in percentage of all female or male participants respectively. We used chi square testing to compare the characteristics between male and female participants.

In these 5266 subjects a total of 308 lesions had a clinical diagnosis of skin malignancy: 38 were suspicious for lentigo maligna, 34 for melanoma, 173 for basal cell carcinoma, 32 for squamous cell carcinoma and in 31 cases another malignant lesion was suspected, details of clinically diagnoses are shown in Table 2. In 3386 subjects (64.3%) no further investigations were deemed required (= shown skin lesion not suspicious for skin malignancy); in 968 (18.4%) a clinical follow-up assessment and in 764 (14.5%) an excision or biopsy was recommended.

**Table 2:** **Clinical classification of lesions**

**Clinical classification**	**N**	%
Dermal nevus	1541	29.26%
Seborrhoic wart	1224	23.24%
Angioma	324	6.15%
other, benign	818	15.53%
Actinic keratosis	426	8.09%
Atypic nevus	450	8.55%
Lentigo maligna	38	0.72%
Melanoma	34	0.65%
Basal cell carcinoma	173	3.29%
Squamous cell carcinoma	32	0.61%
Other, malignant	31	0.59%
Not stated	175	3.32%
Total	**5266**	**100**%

For every participant only one lesion was documented.

Detailed results of the multiple logistic regression model concerning the probability of a clinical malignant diagnosis are shown in Table 3. We found significant influencing factors were age, sex, reason for participation and skin phototype. The results of the second multiple regression model concerning further need of investigation (either biopsy/excision or clinical control) is shown in Table 4. Participants with more than 100 naevi and a positive family history were more likely to get a recommendation for further investigations. To assess the risk factors where dermatologists recommended further management even though the clinical diagnosis was benign, we created a multiple regression model.

**Table 3:** **Multiple regression analysis**, **outcome clinical malignancy**

	**Odds ratio**	**Standard error**	**Significance level**
Sex	0.691	0.098	0.009
Age	1.049	0.005	<0.001
Number of Naevi	1.170	0.261	0.482
Skin phototype	0.629	0.066	<0.001
Reason for participation	1.658	0.235	<0.001
Family history	0.637	0.225	0.201

Multiple logistic regression analysis regarding influential factors concerning clinical malignancy. Categories, reference value is indicated by a *(Sex 0 = male*, 1 = female; age in years, 1 = *; Number of Naevi 0 = less than 100*, 1 = more than 100; skin phototype Fitzpatrick types 1*-6; reason for participation 1 = self-perceived skin abnormality 0 = free skin checkup* and other reasons; family history 1 = positive family history for skin cancer, *0 = no skin cancer in family). Participants with missing data were excluded from this analysis, a total of 4360 entries could be included in this model, R2 of this model = 0.11.

**Table 4:** **Multiple regression analysis management**

	**Odds ratio**	**Standard error**	**Significance level**
Number of naevi	3.583	0.354	<0.001
Family history	1.406	0.191	0.012
Skin phototype	0.803	0.040	<0.001
Age	1.014	0.002	<0.001

Influence factors where further management was recommended despite a bening clinical skin lesion. Reference value is indicated with * (Naevi: *0 = less than 100 Naevi, 1 = more than 100 Naevi; Family history of skin cancer *0 = no, 1 = positive family history; skin phototype according to phototype 1 as reference; age in years). Participants with clinical malignant diagnosis or missing data were excluded from the analysis. 4062 entries were included in this model; R2 of this model was 0.0493.

## Discussion

The attendance at the SSCD to show a self-perceived suspicious skin lesion was the strongest predictor in our multiple regression model (OR 1.6) for a clinical diagnosis of skin malignancy. The national skin cancer campaign aims at raising awareness for skin cancer, promoting sun protection and emphasising the importance of early detection. This aim alone might already legitimate a prevention campaign such as the SSCD. An easy access to a specialist in case of a self-perceived abnormality of a skin lesion might therefore be very helpful. Still whether SSCD is the right instrument is questionable, doubts on safety and quality regarding a single-lesion examination and no routine use of dermoscopy remain and needs to be assessed in future SSCD campaigns.

The uncertainty and limitations of the SSCD might also be reflected in the discrepancy between the number of clinical malignant diagnoses (5.8%) and the number of participants, where a further workup (clinical observation or excision/biopsy) was recommended (32.9%). We investigated possible reasons for this discrepancy in our multiple regression model; participants with risk factors were more likely to get a recommendation for further workup, which could be explained by the context of the SSCD, since participants were only allowed to show one skin lesion. While this skin lesion was possibly benign, the dermatologists nevertheless could have recommended a dermatological consultation since he was not able to conduct a complete dermatological workup. This could raise awareness concerning the risk factors for skin cancer and the importance of regular control in participants with more risk factors, but the direct benefit of the skin lesion screening remains questionable. For clinics and private practices the event might also be an incentive to attract future patients and therefore be a useful public relations event; in case of a clinical uncertainty this might also contribute to recommend a clinical follow up rather than a reassurance.

Former studies have shown skin self-examination (SSE) to be effective in high-risk patients [15-18]. Our study suggests SSE might also work in a general population. From a health services perspective this results implies further research should focus on possibilities to allow an easier access to a specialist, in case of self-perceived skin lesion, especially outside such events. This also might reduce system delay from diagnosis to treatment [31].

Our analysis concerning clinical diagnosis of skin malignancy revealed an unexpected result regarding a positive family history of skin cancer as a risk factor. Participants with a positive family history had a lower risk of a clinical diagnosis of skin malignancy than participants without. Even though this result was statistically not significant in our multiple logistic regression model, this finding raises some questions about the study population. In our study 7.2% stated to have a family history for skin cancer. In the evaluation of the Italian Euromelanoma campaign in the years 2005–2007 7% of the participants recalled a history of skin cancer and 5.2% of melanoma in a family member [25]. The percentage was higher in the Swedish Euromelanoma population, where 14.8% indicated a family history of melanoma [24]; in the Swedish Euromelanoma population the skin phototype were 32.9% with type II and 58.3% type III skin phototype, compared to 44.7% type II and 33.0% type III in our population. One possible reason for this finding could therefore be an incomplete reporting of family history in the participants’ questionnaire and the result might be biased. Another reason could be that persons with family history were possibly already under regular skin control and therefore did not participate at the SSCD.

### Strength and limitations

In our study with the complete dataset on subjects participating on the “Swiss Skin Cancer Day” in 2012, we could show that SSE seems to ameliorate effectiveness of screening and raise the detection rate of clinical diagnoses of skin malignancies. These results are limited by the fact than only single lesions were assessed during the event. Furthermore in our study we were only able to include clinical diagnosis and could not confirm the diagnoses either by dermoscopy or histological findings, this could possibly lead to an overdiagnosis. Also recommendations for a clinical control and/or an excision of the skin lesions were in very high, we do not know what the reasons are for that high number and how many patients really participated in a follow up.

## Conclusion

A self-perceived suspicious lesion was the strongest predictor for a clinical diagnosis of skin malignancy at the SSCD. This suggests that skin self-examination might also work in general population. Future research should focus on better access to a specialist in case a suspicious skin lesion was discovered. Safety and quality of the SSCD should be further investigated, especially concerning the discrepancy between the low number of malignant lesions and the high quantity of participants where further clinical examinations or interventions were recommended.

## Additional file

Additional file 1:
**Questionnaire of the Swiss Skin Cancer Day 2012.**

## References

[1] de Vries E, Coebergh JW (2004). Cutaneous malignant melanoma in Europe. Eur J Cancer.

[2] BFS SFSON, Switzerland) (2013). Epidemiology of Cancer: Melanoma accessed online June 10th 2013 via.

[3] Ferlay J, Steliarova-Foucher E, Lortet-Tieulent J, Rosso S, Coebergh JWW, Comber H, Forman D, Bray F (2013). Cancer incidence and mortality patterns in Europe: Estimates for 40 countries in 2012. Eur J Cancer.

[4] Balch CM, Gershenwald JE, Soong SJ, Thompson JF, Atkins MB, Byrd DR, Buzaid AC, Cochran AJ, Coit DG, Ding S, Eggermont AM, Flaherty KT, Gimotty PA, Kirkwood JM, McMasters KM, Mihm MCJ, Morton DL, Ross MI, Sober AJ, Sondak VK (2009). Final version of 2009 AJCC melanoma staging and classification. J Clin Oncol.

[5] Freedberg KA, Geller AC, Miller DR, Lew RA, Koh HK (1999). Screening for malignant melanoma: A cost-effectiveness analysis. J Am Acad Dermatol.

[6] Geller AC, Swetter SM, Oliveria S, Dusza S, Halpern AC (2011). Reducing mortality in individuals at high risk for advanced melanoma through education and screening. J Am Acad Dermatol.

[7] Choudhury K, Volkmer B, Greinert R, Christophers E, Breitbart EW (2012). Effectiveness of skin cancer screening programmes. Br J Dermatol.

[8] Geller AC, Greinert R, Sinclair C, Weinstock MA, Aitken J, Boniol M, Capellaro M, Dore JF, Elwood M, Fletcher SW, Gallagher R, Gandini S, Halpern AC, Katalinic A, Lucas R, Marghoob AA, Nolte S, Schuz J, Tucker MA, Volkmer B, Breitbart E (2010). A nationwide population-based skin cancer screening in Germany: proceedings of the first meeting of the International Task Force on Skin Cancer Screening and Prevention (September 24 and 25, 2009). Cancer Epidemiol.

[9] Helfand M, Mahon SM, Eden KB, Frame PS, Orleans CT (2001). Screening for skin cancer. Am J Prev Med.

[10] Katalinic A, Waldmann A, Weinstock MA, Geller AC, Eisemann N, Greinert R, Volkmer B, Breitbart E (2012). Does skin cancer screening save lives?: An observational study comparing trends in melanoma mortality in regions with and without screening. Cancer.

[11] Wolff T, Tai E, Miller T (2009). Screening for skin cancer: an update of the evidence for the U.S. Preventive Services Task Force. Ann Intern Med.

[12] De Giorgi V, Grazzini M, Rossari S, Gori A, Papi F, Scarfi F, Savarese I, Gandini S (2012). Is Skin Self-Examination for Cutaneous Melanoma Detection Still Adequate?. A Retrospective Study Dermatol.

[13] Breitbart EW, Waldmann A, Nolte S, Capellaro M, Greinert R, Volkmer B, Katalinic A (2012). Systematic skin cancer screening in Northern Germany. J Am Acad Dermatol.

[14] Eisemann N, Waldmann A, Katalinic A (2014). [Incidence of melanoma and changes in stage-specific incidence after implementation of skin cancer screening in Schleswig-Holstein]. Bundesgesundheitsblatt Gesundheitsforschung Gesundheitsschutz.

[15] Pollitt RA, Geller AC, Brooks DR, Johnson TM, Park ER, Swetter SM (2009). Efficacy of skin self-examination practices for early melanoma detection. Cancer Epidemiol Biomarkers Prev.

[16] Swetter SM, Pollitt RA, Johnson TM, Brooks DR, Geller AC (2012). Behavioral determinants of successful early melanoma detection: role of self and physician skin examination. Cancer.

[17] Titus LJ, Clough-Gorr K, Mackenzie TA, Perry A, Spencer SK, Weiss J, Abrahams-Gessel S, Ernstoff MS (2013). Recent skin self-examination and doctor visits in relation to melanoma risk and tumour depth. Br J Dermatol.

[18] Weinstock MA, Risica PM, Martin RA, Rakowski W, Dube C, Berwick M, Goldstein MG, Acharyya S, Lasater T (2007). Melanoma early detection with thorough skin self-examination: the “Check It Out” randomized trial. Am J Prev Med.

[19] Badertscher N, Senn O, Rossi PO, Wensing M, Rosemann T, Tandjung R (2013). [Skin cancer in primary care: frequency, need to further education and subjective diagnostic certainty. A cross sectional survey among general practitioners in Canton of Zurich]. Praxis (Bern 1994).

[20] Heinzerling LM, Dummer R, Panizzon RG, Bloch PH, Barbezat R, Burg G, League SC (2002). Prevention campaign against skin cancer. Dermatology.

[21] Bulliard JL, Maspoli M, Panizzon RG, Hohl D, Gueissaz F, Levi F (2008). Evaluation of the Euromelanoma skin cancer screening campaign: the Swiss experience. J Eur Acad Dermatol Venereol.

[22] Bulliard JL, Panizzon RG, Levi F (2009). [Epidemiology of epithelial skin cancers]. Rev Med Suisse.

[23] Stratigos AJ, Forsea AM, van der Leest RJ, de Vries E, Nagore E, Bulliard JL, Trakatelli M, Paoli J, Peris K, Hercogova J, Bylaite M, Maselis T, Correia O, Del Marmol V (2012). Euromelanoma: a dermatology-led European campaign against nonmelanoma skin cancer and cutaneous melanoma. Past, present and future. Br J Dermatol.

[24] Paoli J, Danielsson M, Wennberg AM (2009). Results of the ‘Euromelanoma Day’ screening campaign in Sweden 2008. J Eur Acad Dermatol Venereol.

[25] Seidenari S, Benati E, Ponti G, Borsari S, Ferrari C, Albertini G, Altomare G, Arcangeli F, Aste N, Bernengo MG, Bongiorno MR, Borroni G, Calvieri S, Chimenti S, Cusano F, Fracchiolla C, Gaddoni G, Girolomoni G, Guarneri B, Lanzoni A, Lombardi M, Lotti T, Mariotti A, Marsili F, Micali G, Parodi A, Peris K, Peserico A, Quaglino P, Santini M (2012). Italian Euromelanoma Day Screening Campaign (2005–2007) and the planning of melanoma screening strategies. Eur J Cancer Prev.

[26] van der Leest RJT, de Vries E, Bulliard JL, Paoli J, Peris K, Stratigos AJ, Trakatelli M, Maselis TJEML, Situm M, Pallouras AC, Hercogova J, Zafirovik Z, Reusch M, Olah J, Bylaite M, Dittmar HC, Scerri L, Correia O, Medenica L, Bartenjev I, Guillen C, Cozzio A, Bogomolets OV, del Marmol V (2011). The Euromelanoma skin cancer prevention campaign in Europe: characteristics and results of 2009 and 2010. J Eur Acad Dermatol.

[27] AAD AAoD (2012). Free skin cancer screenings, accessed online November 28th 2012.

[28] Hansen RP, Olesen F, Sorensen HT, Sokolowski I, Sondergaard J (2008). Socioeconomic patient characteristics predict delay in cancer diagnosis: a Danish cohort study. BMC Health Serv Res.

[29] Huber CA, Bandi-Ott E, Kaser L, Rosemann T, Senn O (2009). [Medical check-ups on the <<Gesundheitsschiff>>. Benefit for population at risk or self-selection of healthy and health-conscious patients?]. Praxis (Bern 1994).

[30] SAMW SAoMS (2009). Research with humans [Forschung mit Menschen] accessed online June 19th 2013.

[31] Hansen RP, Vedsted P, Sokolowski I, Sondergaard J, Olesen F (2011). Time intervals from first symptom to treatment of cancer: a cohort study of 2,212 newly diagnosed cancer patients. BMC Health Serv Res.

