# Supplementary material for: The role of skin self-examination at the Swiss skin cancer day
Source: BMC Health Serv Res. 2014 Nov 19;14:581. doi: 10.1186/s12913-014-0581-6 (PMC4237758; doi:10.1186/s12913-014-0581-6)
Supplement: Additional file 1: — Questionnaire of the Swiss Skin Cancer Day 2012. [file 12913_2014_581_MOESM1_ESM.docx]

**Questionnaire Swiss Skin Cancer Day May 7^th^ / 9^th^ and 11th 2012**

**A demographic information of participants**

*Sex*

O female

O male

*Age in years: _______________*

*Nationality: _______________*

*Reason for participation*

O Checkup

O self-perceived suspiocous skin lesion

O skin cancer of friends

O sent by partner

O other reason

O Family history of skin cancer

**B skin lesion**

*Skin type (I to VI): _______________________*

*Number of Naevi (estimated):*

O less than 100

O more than 100

*Clinical classifcation*

O dermal naevus

O seborrhoic wart

O angioma

O orther, benign lesion

O actinic keratosis

O atpyic naevus

O lentigo maligna

O melanoma

O basal cell carcinoma

O squamous cell carcinoma

O other malignant

**C management**

O harmless (no further step necessary)

O clinical observation recommended

O excision or biopsy necessary

**Remark: Original version was German and French, translated for adnex publication purposes**.
